# Understanding the Superior Stability of Single‐Molecule Magnets on an Oxide Film

**DOI:** 10.1002/advs.201901736

**Published:** 2019-09-30

**Authors:** Michał Studniarek, Christian Wäckerlin, Aparajita Singha, Romana Baltic, Katharina Diller, Fabio Donati, Stefano Rusponi, Harald Brune, Yanhua Lan, Svetlana Klyatskaya, Mario Ruben, Ari Paavo Seitsonen, Jan Dreiser

**Affiliations:** ^1^ Swiss Light Source Paul Scherrer Institut (PSI) CH‐5232 Villigen Switzerland; ^2^ Institute of Physics (IPHYS) École Polytechnique Fédérale de Lausanne (EPFL) Station 3 CH‐1015 Lausanne Switzerland; ^3^ Institute of Physics The Czech Academy of Sciences Cukrovarnická 10 CZ‐162 00 Prague 6 Czech Republic; ^4^ Center for Quantum Nanoscience Institute for Basic Science (IBS) 03760 Seoul Republic of Korea; ^5^ Department of Physics Ewha Womans University 03760 Seoul Republic of Korea; ^6^ Institute of Nanotechnology (INT) Karlsruhe Institute of Technology (KIT) Hermann‐von‐Helmholtz‐Platz 1 D‐76344 Eggenstein‐Leopoldshafen Germany; ^7^ Institut de Physique et Chimie des Matériaux de Strasbourg (IPCMS) Centre National de la Recherche Scientifique (CNRS) Université de Strasbourg 23 rue du Loess, BP 43 F‐67034 Strasbourg Cedex 2 France; ^8^ Département de Chimie École Normale Supérieure F‐75005 Paris France; ^9^ Centre National de la Recherche Scientifique (CNRS) Paris Sciences et Lettres Sorbonne Université F‐75005 Paris France

**Keywords:** molecular spintronics, single‐ion magnets, single‐molecule magnets, surfaces, X‐ray absorption spectroscopy

## Abstract

The stability of magnetic information stored in surface adsorbed single‐molecule magnets is of critical interest for applications in nanoscale data storage or quantum computing. The present study combines X‐ray magnetic circular dichroism, density functional theory and magnetization dynamics calculations to gain deep insight into the substrate dependent relevant magnetization relaxation mechanisms. X‐ray magnetic circular dichroism reveals the opening of a butterfly‐shaped magnetic hysteresis of DyPc_2_ molecules on magnesium oxide and a closed loop on the bare silver substrate, while density functional theory shows that the molecules are only weakly adsorbed in both cases of magnesium oxide and silver. The enhanced magnetic stability of DyPc_2_ on the oxide film, in conjunction with previous experiments on the TbPc_2_ analogue, points to a general validity of the magnesium oxide induced stabilization effect. Magnetization dynamics calculations reveal that the enhanced magnetic stability of DyPc_2_ and TbPc_2_ on the oxide film is due to the suppression of two‐phonon Raman relaxation processes. The results suggest that substrates with low phonon density of states are beneficial for the design of spintronics devices based on single‐molecule magnets.

## Graphical abstract

DyPc_2_ single‐molecule magnets on magnesium oxide exhibit a significantly more stable magnetic moment than on a silver substrate. Modeling of the magnetic hysteresis loops reveals that the stabilization effect originates from the suppression of the two‐phonon Raman process. The results suggest the perspective of enhancing the magnetic stability by weakly adsorbing substrates with low density of vibrational modes.

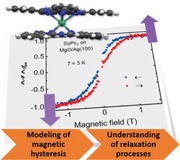

## 1 Introduction

**Figure 1:**
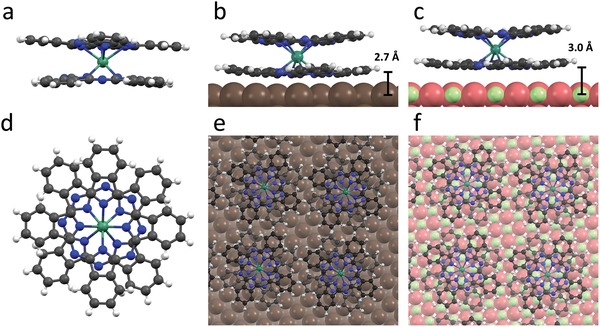
Structure of bulk and surface‐adsorbed LnPc_2_. Ball‐and‐stick model of an LnPc_2_ molecule^63^ in a) side and d) top view. Most stable adsorption conformations of YPc_2_ b,e) on Ag(100) and c,f) on MgO(5 ML)/Ag(100). Color code: carbon: dark gray; nitrogen: blue; hydrogen: light gray; yttrium/dysprosium/terbium: turquoise; silver: brown; magnesium: green; oxygen: red.

Surface‐adsorbed single‐molecule magnets (SMMs)^1^, ^2^, ^3^, ^4^, ^5^, ^6^, ^7^, ^8^, ^9^ offer a unique combination of nanoscale dimension, slow magnetization dynamics, monodispersity, and quantum behavior. Because of these properties they are attractive candidates for applications in spintronics devices,^10^, ^11^ nanoscale addressable memory cells,^2^ or for the implementation of quantum computing.^12^, ^13^, ^14^ The last years have seen tremendous progress in the synthesis of organometallic SMMs culminating in the recent demonstration of magnetic hysteresis up to ≈80 K in the bulk phase,^15^ for the first time lying above the technologically important threshold of liquid nitrogen temperature. However, despite the demonstration of ground breaking results on isolated SMM devices,^11^, ^16^ the realization of large scale addressable arrays on planar surfaces exhibiting magnetic remanence at liquid nitrogen temperature still remains elusive. The reason is the molecule‐surface interaction, which is often highly detrimental for the unique magnetic properties of SMMs.^17^, ^18^, ^19^, ^20^, ^21^, ^22^, ^23^, ^24^, ^25^ Therefore, despite the existence of high‐temperature SMMs, the magnetic properties of surface‐adsorbed SMMs are in most cases limited by the poorly understood relaxation mechanisms involving the substrate. Because of their chemical robustness and their flat adsorption on planar surfaces, the isostructural lanthanide (Ln) double decker SMMs TbPc_2_ and DyPc_2_ 26, 27, 28 (**Figure** **1**) are most frequently used as model systems. In all previous cases, in which a butterfly shaped magnetic hysteresis opening was obtained on LnPc_2_ SMMs in the sub‐monolayer range, the molecules were adsorbed on weakly interacting substrates such as gold,^20^ graphite,^21^, ^22^ or graphene.^24^, ^25^ Alternatively, molecular functionalization was applied in order to reduce the interaction by spatial separation from the substrate.^2^, ^23^, ^29^ Recently, some of the present authors reported that a thin magnesium oxide film massively stabilizes the magnetic ground states of TbPc_2_ SMMs across a field range of several Tesla, resulting in a significant remanence and a wide hysteresis opening^30^ besides promoting magnetic remanence of adsorbed Ho atoms.^31^ The results on LnPc_2_ and Fe_4_ SMMs^2^, ^32^ suggest the importance of the physisorption regime, i.e., the regime of weak molecule‐surface interaction and low hybridization. In addition it was hypothesized that the insulation from conduction electrons of the underlying substrate by the insulating tunnel barrier and thus the suppression of spin‐flip scattering processes,^30^, ^31^ as well as the high stiffness of the oxide film hindering spin‐phonon relaxation^31^ play a role. Despite this considerable progress, experimental evidence of the dynamics of other SMMs on oxide surfaces and the knowledge about the key magnetization relaxation mechanisms are required.

Here, we experimentally study DyPc_2_ molecules (cf. Figure 1) deposited as a sub‐monolayer (ML) on a thin film of MgO on Ag(100) and on bare Ag(100) to gain the insight into various magnetic relaxation pathways and unravel the role of oxide decoupling layer in the system. We use X‐ray absorption spectroscopy (XAS) and X‐ray magnetic circular dichroism^33^ (XMCD), which yields the element specific magnetic moment as well as the magnetic anisotropy with sub‐monolayer sensitivity. While in the bulk the TbPc_2_ molecule exhibits the slowest magnetization dynamics within the LnPc_2_ family, our choice of DyPc_2_ for the present study allows to study a structurally similar, but faster relaxing system. DyPc_2_ is virtually isostructural to TbPc_2_ with close to perfect antiprismatic (*D*_4d_) symmetry and nearly identical Ln ion radii (1.04 vs 1.03 Å for Tb^3+^ and Dy^3+^, respectively).^34^ The essential difference between these molecular species lies in the effective energy barrier for magnetization reversal, which is one order of magnitude smaller in DyPc_2_.^28^ The experimental data gathered on surface adsorbed DyPc_2_ and TbPc_2_ SMMs provides us with sufficient evidence to scrutinize the role of the oxide surface on the stabilization of the SMMs' magnetization dynamics.

## 2 Results

### 2.1 Density Functional Theory

The most stable adsorption conformations of LnPc_2_ on MgO(5 ML)/Ag(100) and bare Ag(100), as obtained from density functional theory (DFT) calculations, are depicted in Figure 1. To reduce the already significant computational cost and to circumvent the potential shortcomings of the applicable approximations of the exchange‐correlation function on the electronic structure of the lanthanides, the calculations were performed on the isostructural but diamagnetic and chemically identical YPc_2_. On Ag(100) and MgO(5 ML)/Ag(100) the most stable adsorption sites are the ones where the Y atom is centered above the Ag(100) hollow site, and above the oxygen site, respectively. The molecules form square superlattices on both substrates (intermolecular distance 14.5 Å) with lattice vectors rotated by ±8° away from the [001] and [010] in‐plane crystallographic directions of the substrates, consistent with previous STM investigations.^30^ In both cases the molecules lie flat with the phthalocyanine ligands quasi‐parallel to the surface plane. On both surfaces a slight bending of the phthalocyanine ligands away from the surface is observed. The adsorption heights, as determined from the average height of the N atoms in the lower phthalocyanine sheet above the substrate atoms, are 2.66 and 2.98 Å for the cases of Ag and MgO, respectively, suggesting weak adsorption and low hybridization between molecular and surface atomic orbitals. For comparison, on the more reactive Ni(111) surface, an adsorption height of 1.97 Å was previously found,^35^ and 3 Å on graphene/Ni(111). In the present case the heights are consistent with only a small variation of the adsorption energy of *E*_ads,Ag_ = −4.92 eV and *E*_ads,MgO_ = −4.79 eV. DFT calculations for CoPc/Au(111)^36^ yield values of ≈−4.8 eV and an adsorption height of ≈3 Å, and experimentally an adsorption energy of −3.2 eV for CoPc/graphene/Ir(111) was measured.^37^ In view of the larger double decker molecules compared to the “single decker” CoPc and of the importance of the van der Waals contribution, the adsorption height of ≈3 Å and the adsorption energy of −4.79 eV for YPc_2_/MgO/Ag obtained from DFT in our work falls indeed into the range of weakly interacting systems. The adsorption energies of different other conformations are given in Table S1 in the Supporting Information. Finally, the movement‐of‐charge patterns for the Ag(100) and the MgO(5 ML)/Ag(100) substrates upon molecule adsorption were evaluated (cf. Figure S1 in the Supporting Information). The similarity of these patterns, together with the fact that the ligand hole, i.e., the radical spin, is absent in TbPc_2_/Ag(111),^38^, ^39^ suggests the absence of the ligand hole in LnPc_2_/MgO(5 ML)/Ag(100). Note that in ref. 30 the difference in hysteresis opening between samples with four and five MLs of MgO is negligibly small, therefore it is expected that the DFT results are equally valid for the samples with four MLs of MgO. Further details are given in the Supporting Information.

### 2.2 X‐Ray Absorption Spectroscopy and Dichroism

**Figure 2:**
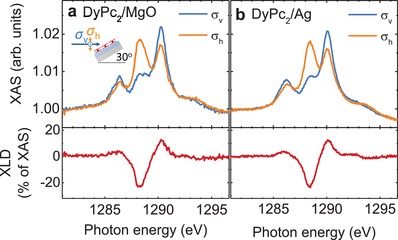
X‐ray linear dichroism of surface adsorbed DyPc_2_. X‐ray absorption spectra recorded at the Dy M_5_ edge with linear vertical (σ_v_) and horizontal (σ_h_) X‐ray polarization (top panel) and corresponding XLD (bottom panel) on DyPc_2_(sub‐ML) on a) MgO(4 ML)/Ag(100) and b) on Ag(100) at a temperature of 2.5 ± 0.5 K. The spectra were recorded at grazing incidence 60° to the sample normal, and at 50 mT magnetic field applied along the X‐ray beam propagation direction.

**Figure** **2** shows the X‐ray absorption spectra and X‐ray linear dichroism (XLD) at the Dy M_5_ edge (3d → 4f transitions) obtained on 0.5 ML DyPc_2_ deposited on MgO(4 ML)/Ag(100) and on Ag(100). For visibility, only the M_5_ edge is shown here, while the complete spectrum is plotted in Figure S2 in the Supporting Information. The XLD spectrum, defined as the difference between absorption spectra acquired with linear vertical (σ_v_) and horizontal (σ_h_) polarization at grazing incidence allows to experimentally resolve the angular distribution of the 4f atomic orbitals and reveals the molecular orientation on the surface. We observe the typical three‐peak structure of Dy^3+^ with a strong XLD confirming the flat orientation of the molecules^22^, ^40^, ^41^ with their phthalocyanine ligands oriented parallel to the surface, in line with the geometry from our DFT calculations and with the scanning tunneling microscopy studies on TbPc_2_.^30^ We found slight variations in the strength of the XLD spectra between different DyPc_2_/MgO/Ag samples that we ascribe to small differences in the roughness of the MgO film. Representative spectra are shown in Figure 2a while the XLD of the sample on which we acquired the XMCD and the magnetic hysteresis loops shown below is plotted in Figure S3 in the Supporting Information.

**Figure 3:**
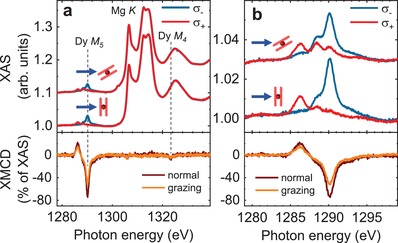
X‐ray magnetic circular dichroism of surface‐adsorbed DyPc_2_. XAS (top panels) and corresponding XMCD (bottom panels) acquired on DyPc_2_(sub‐ML)/MgO(4 ML)/Ag(100) at a) Dy M_4,5_ edges of DyPc_2_ with b) zoom on the Dy M_5_ edge only. The Dy M_4_ edge is dominated by the Mg K edge. The spectra were recorded at a temperature of 2.5 ± 0.5 K in normal (0°) and grazing (60°) X‐ray incidence, and at a field of 6.8 T applied parallel to the X‐ray beam.

We used XMCD to access the magnetic properties of the Dy ions in the double decker molecules on the MgO thin film. This technique reveals the magnetic moment residing in the Dy 4f shell projected onto the incident beam direction. The Dy 4f magnetic moment constitutes virtually the whole magnetic moment of the molecule because the radical spin is absent in the present samples and the nuclear magnetic moment and hybridization effects of the inner 4f shell with ligand or surface atomic orbitals are negligibly small. **Figure** **3** depicts XAS and XMCD spectra at the Dy M_4,5_ edges at normal and grazing incidence. The measurements reveal a larger amplitude of the normal XMCD signal evidencing an out‐of‐plane easy axis of magnetization of the adsorbed molecules. This is a consequence of the strong easy‐axis anisotropy of the Dy^3+^ ion in the sandwich‐type ligand field, which repels the oblate Dy 4f charge distribution axially from the ion,^42^ and the flat orientation of DyPc_2_ on the surface.^4^, ^30^ The XAS and XMCD of DyPc_2_ adsorbed on the bare Ag(100) surface (Figure S4, Supporting Information) are virtually identical. The similarity of the X‐ray spectra on MgO and on Ag(100) indicates that the molecules are adsorbed in the same flat geometry on both substrates and that their magnetic anisotropy is preserved, ruling out a different molecular orientation as the reason for the different dynamic magnetic properties. The extraction of the magnetic moment of Dy from XMCD by the sum rule formalism was not possible due to the dominant Mg K edge background.

**Figure 4:**
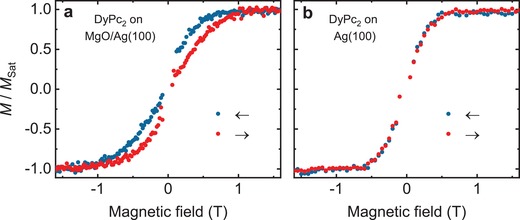
Magnetic hysteresis of DyPc_2_. Hysteresis loops from XMCD at the Dy M_5_ edge of a) DyPc_2_(sub‐ML)/MgO(4 ML)/Ag(100) and b) DyPc_2_(sub‐ML)/Ag(100). The data were recorded at normal X‐ray incidence at 2 T min^−1^ magnetic field sweep rate and at a temperature of 2.5 ± 0.5 K.

Magnetic hysteresis loops acquired using XMCD on DyPc_2_/MgO evidence a butterfly‐shaped hysteresis with a 1 T‐wide opening (**Figure** **4**a), while the loop is closed when the molecules are adsorbed directly on Ag(100) (Figure 4b). This points to a sizable oxide‐layer induced slowdown of the magnetization dynamics and to an increased blocking temperature of DyPc_2_/MgO in comparison to the previous study on HOPG.^22^ Note, that in contrast to TbPc_2_, the slowdown effect is observed mainly at *µ*_o_ *H* ≠ 0. The fact that the molecules show very small or no remanence suggests strong quantum tunneling of magnetization (QTM; see below).^22^

### 2.3 Modeling of the Magnetization Dynamics

In order to extract quantitative information from the magnetic hysteresis loops a rate equation model is employed, which is solved numerically. Details of the numerical solution are given in the Experimental Section. The model allows to calculate the time evolution of the magnetic moment of the LnPc_2_ SMMs. It takes into account the time dependent applied magnetic field as well as the temperature and field dependencies of the different magnetic relaxation processes. We have applied the model to the present experimental data obtained on DyPc_2_ along with the one obtained earlier on TbPc_2_ in order to derive a broad picture of the relevant relaxation mechanisms.

The total magnetic moment of bulk LnPc_2_ in its neutral form, as employed in the experiments, is the sum of the contributions from the Ln electronic spin *J*, the radical (ligand hole) spin *S* and the nuclear spin *I*. As mentioned before, DFT calculations (Supporting Information) indicate that on MgO and on the bare silver surface a substrate‐molecule charge transfer occurs leading to the suppression of the radical spin, consistent with previous experimental^38^ and theoretical^35^ studies. Therefore, in the present case the magnetic moment of the LnPc_2_ originates, to a very good approximation, from the electronic spin *J* only. The XMCD technique is sensitive to this electronic spin. Taking into account the coupled electronic *J* and nuclear *I* spins indicates that TbPc_2_ on both Ag and MgO has half‐integer spin (*J*_Tb_ = 6, *I*_Tb_ = 3/2). In contrast, 56% of the Dy atoms have *I*_Dy_ = 0 and 44% have *I*_Dy_ = 5/2 following the natural abundance of Dy isotopes. The electronic spin of Dy is *J*_Dy_ = 15/2 in all cases. Therefore, about equal fractions of the DyPc_2_ molecules possess half‐integer and integer total spins, respectively.

The time dependent magnetic moment *M*(*t*) of an SMM at a fixed temperature *T* and at varying magnetic field *H*(*t*) is given by

(1) $$\frac{dM}{dt}=-\Gamma \left(H\left(t\right),T\right)\left[M\left(H\left(t\right)\right)-M_{\mathrm{eq}}\;\left(H\left(t\right),T\right)\right]$$

Here, the total relaxation rate $\Gamma \;(H(t),T)=\sum \tau_{i}^{-1}{\;}^{}(H(t),T)$ is the sum of the rates arising from different relaxation processes dependent on the temperature and on the applied magnetic field. Due to the relaxation, the magnetic moment of the probed ensemble of molecules decays exponentially to the equilibrium value *M*_eq_(*H*).^43^ In the present case *M*_eq_(*H*) is calculated from a spin‐Hamiltonian model taking into account published Stevens parameters for DyPc_2_ and TbPc_2_.^44^ Physically, the field‐dependent magnetic relaxation rates determine the detailed shape of the magnetic hysteresis loops, and our model allows to decode quantitatively the individual contributions of the magnetic relaxation processes by a rather inexpensive numerical calculation.

In our studies we considered the effects of different relaxation processes arising from spin–phonon coupling, quantum tunneling of magnetization,^43^ X‐ray induced magnetic demagnetization,^30^, ^45^ and spin‐flip scattering processes^46^, ^47^ induced by substrate conduction electrons. The latter two processes will be discussed at the end of this Section. Spin–phonon coupling results, in first and second order, in a one‐phonon “direct” spin relaxation process and in two‐phonon Raman and Orbach processes.^48^ While in the recent literature, the details of the Raman process related to spin–phonon coupling are discussed controversially,^49^, ^50^ we stick here to the formulae as derived formerly by Orbach.^48^ In the direct process one phonon is absorbed or emitted by the spin, and the corresponding relaxation rate is given by^48^

(2) $$\Gamma_{\mathrm{direct}}=\;AH^{m}\coth\left(g_{J}\mu_{B}\mu_{0}m_{j}H/k_{B}T\right)$$

Here, the *A* parameter contains the response of the ligand‐field potential to local vibrations and material specific parameters such as the sound velocity and the phonon density of states.^8^, ^48^ *H* is the applied magnetic field, *g*_J_ is the Landé *g*‐factor (*g*_J,Dy_ = 4/3 and *g*_J,Tb_ = 3/2), *m*_j_ is the *z*‐projection of the total angular momentum quantum number of the ground state (*m*_j,Dy_ = 13/2 and *m*_j,Tb_ = 6), *µ*_B_ denotes the Bohr magneton, and *k*_B_ the Boltzmann constant. The exponent assumes *m* = 5 and *m* = 3 for half‐integer spin (Kramers ions) and integer spin values (non‐Kramers ions), respectively.^48^ Consequently, for TbPc_2_ we used *m* = 5, and in the case of DyPc_2_ two hysteresis loops were calculated with *m* = 3 and *m* = 5 and superposed with weighting factors according to the natural abundance of the isotopes. For Dy, the same *A* parameter was used in Equation (2) independent of the Kramers nature to avoid overparameterization of the model. The ratio of *A* parameters for the Kramers and non‐Kramers cases is estimated (using Equations (20) and (21) in ref. 8) to be *A*_nK_/*A*_K_ ≈ 2 in the case of µ_0_ *H* = 1 T and for the separation between ground and first excited doublet of Δ_12_ ≈ 30 cm^−1^ (cf. ref. 27). Because of the rather similar values of *A*_nK_ and *A*_K_ and, again, to avoid overparameterization of the model only one *A* parameter was allowed to vary freely in the fits.

The two‐phonon Raman processes as originally described by Orbach exhibit strong temperature dependence.^48^ Furthermore, its magnetic field dependence is determined by the Kramers nature of the spin. For non‐Kramers and Kramers ions the rates are given by^48^ Γ_Ram,nK_ = *R*_r_ *T* ^7^ and $\Gamma_{\mathrm{Ram},K}=R_{r}\;T^{9}+R\prime_{r}^{}H^{2}T^{7}$. In order to avoid overparameterization of our model, we have reduced the complexity down to the simple expression

(3) $$\Gamma_{\mathrm{Ram}}=\;C|H{|}^{l}\;f\left(T\right)$$

The *C* parameter includes the spin–phonon coupling and other material specific quantities.^48^ The magnetic field dependence contained in the exponent *l* and the temperature dependence *f*(*T*) vary in the literature.^50^, ^51^, ^52^, ^53^, ^54^ Because of the constant temperature in the present experiments we have set *f*(*T*) = 1 for simplicity and to maintain a maximum of transparency in the interpretation of the experimental results.

In addition the Orbach process, which also results from spin–phonon coupling, was taken into account using parameter values published in the literature,^27^ however, the effect on the shape of the magnetic hysteresis loops in this work was found to be negligible. Details are given in the Supporting Information.

QTM leads to the acceleration of magnetization relaxation at specific magnetic field‐dependent resonances,^55^, ^56^ at which energy levels become quasi‐degenerate. QTM is promoted by weak distortions of the ligand field acting on the Ln^3+^ ion, which result in a departure from *D*_4d_ symmetry. The QTM rate is expressed as^57^

(4) $$\Gamma_{\mathrm{QTM}}=\frac{B_{1}}{1+B_{2}{\left(H-H_{\mathrm{QTM}}\right)}^{2}}$$

*B*_1_ and *B*_2_ are free parameters and determine the amplitude and the inverse width of the transition peak, respectively. *H*_QTM_ denotes the magnetic field value at which the energy level anti‐crossings occur. Naturally, QTM is efficient in the vicinity of zero field. Note, that the exact QTM peak structure is known to be more complex due to the hyperfine interaction.^55^ However, since we did not observe a significant improvement in the fits to the data the model includes a single QTM peak only for the sake of simplicity.

In addition, the effects of X‐rays and of the substrate conduction electrons on the magnetic relaxation rates were considered. The X‐rays used to measure the magnetic hysteresis loops are known to demagnetize the SMMs with a rate proportional to the X‐ray flux.^30^, ^45^ X‐ray induced demagnetization is taken into account by including a fixed relaxation rate of *Γ_X_* = 10^−3^ s^−1^ based on the previous studies.^30^, ^45^ Finally, the Ag conduction electrons can lead to spin‐flip scattering,^46^, ^47^ in which the spin of the SMM is relaxed by a virtual electron exchange of the SMM with the substrate conduction band combined with a spin flip. This hopping process is expected to proceed through the radical spin, because it is more strongly interacting with the substrate than the Ln^3+^ ion.^35^ Since the magnetization loops of TbPc_2_ on Au^20^ and on Ag are nearly identical, whereas the radical spin is present in the case of Au while it is not on Ag, the spin‐flip scattering process appears to be irrelevant in this system.

Fits were performed by varying the *A* and *C* parameters in Equations (2) and (3) as well as the QTM parameters *B*_1_ and *B*_2_ in Equation (4). The fits were carried out in a hierarchical manner in order to obtain a consistent picture for all studied systems. More specifically, the behavior of TbPc_2_ with and without the MgO film was corroborated first, and the DyPc_2_ results were then fitted based on the same physical interpretation extracted from the TbPc_2_ results. This procedure was chosen because it was not always possible to determine all parameters uniquely. In particular the closed magnetization loop of DyPc_2_/Ag represents a challenge, as only lower bounds of the field dependent relaxation rates can be derived from it. Relaxation faster than these lower bounds would lead to a similarly closed hysteresis. As part of the hierarchical procedure the parameters which could not be determined unambiguously, and which are thus less relevant in the specific case, were fixed to reasonable values.

In the case of TbPc_2_/MgO a fraction of 20% of fast‐relaxing molecules, which follow the equilibrium magnetization curve, were considered. Without this second species it was impossible to obtain any matching of the fitted curve in the vicinity of zero field, while taking into account all relaxation mechanisms mentioned in the above. Physically, the presence of fast‐relaxing molecules can be understood by the presence of molecules adsorbed on or close to defect sites or step edges.

**Figure 5:**
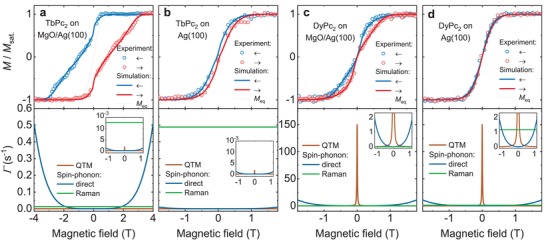
Empirical model of LnPc_2_ magnetization dynamics. (Top panels) Experimental and best‐fit calculated magnetic hysteresis loops for surface adsorbed LnPc_2_ as indicated in the plots. The experimental data shown in panels (a,b) is taken from ref. 30. (Bottom panels) The contributions to the total relaxation rate of the relevant relaxation processes used in the best‐fit calculation. The field sweep rate was 2 T min^−1^ in both experiment and calculation and the temperature was 2.5 ± 0.5 K.

The comparison of the experimental data with the best‐fit simulated hysteresis loops of TbPc_2_ and DyPc_2_ on MgO/Ag(100) and Ag(100) surfaces (**Figure** **5**) reveals excellent agreement between calculations and experiment. In the bottom panels the contributions of the individual magnetic relaxation processes to the total relaxation rate, as extracted from our fits to the experimental data, are shown. In Figure 5c,d the average of the relaxation rates of the direct process, weighted according to the presence of Kramers and non‐Kramers species, is plotted. Note that, as mentioned before, these rates do not differ much in the relevant field range of ≈1 T up to the closing of the magnetic hysteresis. The best‐fit values of the free parameters used to obtain the calculated hysteresis loops shown in Figure 5 are presented in **Table** **1**. In the following the implications of the fits to the experimental magnetization loops will be discussed.

**Table 1:** Best‐fit parameter values used to calculate the magnetic hysteresis loops of surface adsorbed LnPc_2_ as shown in Figure 5. Values in italic were kept fixed during the fits as described in the main text

	Raman	Direct	QTM	
	*C_l_*_= 0_ [s^−1^]	*A* [T^−^ *^m^* s^−1^]	*B*_1_ [s^−1^]	*B*_2_ [T^−2^]
TbPc_2_/MgO	(1.3 ± 0.1) × 10^−2^	(5 ± 1) × 10^−4^	$0.002{\;}_{-0.001}^{+0.002}$	$1.5_{-1.2}^{+1.5}$ × 10^4^
TbPc_2_/Ag	0.5 ± 0.2	*5*	*0.002*	*1.5 × 10^4^*
DyPc_2_/MgO	*0*	0.9 ± 0.3	150 ± 50	$1.5_{-1.2}^{+1.5}\;$× 10^4^
DyPc_2_/Ag	>1.2 ± 0.6	*0.9*	*150*	*1.5 × 10^4^*

## 3 Discussion

First, we focus on the case of TbPc_2_/MgO shown in Figure 5a. A good fit can only be obtained, if QTM, the direct spin‐phonon process and the Raman process with *l* = 0 are included. Regarding the latter a weak field dependence *l* ∈ {1, 2} is still compatible with the experimental data as seen in Figure S5 in the Supporting Information. The bottom panel of Figure 5a reveals that the rate of the QTM and Raman processes at around zero field is the main factor determining the remanence in the TbPc_2_/MgO system. In addition, the waiting time at zero field of ≈20 s needed to change the polarity of the superconducting magnet power supply, which is taken into account in the calculations, has an influence on the shape of the hysteresis loops. The direct spin–phonon process is relevant at high fields and limits the width of the hysteresis. The fact that the rapid closing of the magnetization loop at 3 T is not perfectly reproduced, could arise from shortcomings of the model, which does not take into account the specific phonon density of states of the SMMs and the substrate.

In order to simulate the behavior of TbPc_2_/Ag (cf. Figure 5b), obviously very different magnetic relaxation rates as compared to TbPc_2_/MgO need to be taken into account: The fitting attempts revealed that the hysteresis loop can only be described satisfactorily by increasing the rate of the field independent process by more than one order of magnitude. Varying the rates of QTM and of the direct spin–phonon process turned out to be insufficient to reproduce the experimental data (cf. Figure S6 in the Supporting Information), although an acceleration of these processes on Ag may also be possible. This suggests that QTM and the direct process are not relevant here (cf. Figure 5a (bottom)), but it is unlikely that they are completely absent. Therefore, we have employed fixed *A* and *B*_1,2_ parameters, which were obtained from the fits on TbPc_2_/MgO, as reflected by the parameter values given in italic font in Table 1. Varying the field exponent *l* in Equation (3) yields that a good fit can only be obtained for *l* = 0, i.e., a magnetic‐field independent process. It is worth noting that the field independence of the Raman process was recently suggested also in other, bulk SMMs.^58^

Next, the magnetization dynamics of DyPc_2_ will be discussed (cf. Figure 5c,d). The fits indicate that in DyPc_2_/MgO the magnetic relaxation is dominated by strong QTM at zero field, leading to the butterfly‐type shape without any observable remanence. In analogy to the case of TbPc_2_/MgO also here the strong field dependence of the direct spin–phonon process is responsible for the closing of the magnetic hysteresis loop. However, while in TbPc_2_/MgO this information can be clearly extracted from the fits, it is an assumption for DyPc_2_/MgO and it is difficult to exclude that the Raman process with different field exponents might dominate. Following the hierarchical procedure, the simulation presented in Figure 5d was performed in an analogous way as in the TbPc_2_ experiments. Justified by the same argument given above, the *A* and *B*_1,2_ parameter values were fixed while only varying the *C* parameter resulting in a rate greater than *C_l_*_= 0_ > 1.2 s^−1^. In this case of DyPc_2_ a massive acceleration of QTM and/or of the direct spin‐phonon process on the Ag surface could also lead to the closing of the hysteresis, that is, for DyPc_2_/Ag relaxation rates faster than the ones given in Table 1 would be equally consistent with the experimental data. However, in view of the fit results obtained on the TbPc_2_ magnetic hysteresis, a completely different origin of the DyPc_2_ magnetic behavior compared to TbPc_2_ would be difficult to justify.

Hence the fits to the experimental magnetization loops reveal that the narrowing or closing of the magnetic hysteresis of LnPc_2_ when changing the surface from MgO to Ag are due to a massive acceleration of a process with the field independent or weakly field dependent relaxation rate. This process is associated to the two‐phonon Raman process. While in the case of DyPc_2_ the acceleration of spin relaxation by QTM or by the direct spin‐phonon process could still explain the observed behavior, this is excluded for TbPc_2_ as stated before. The prevalence of the Raman process on Ag(100), in turn, is in agreement with predictions by previous studies of a reduced phonon density of states at MgO with respect to the Ag surface.^31^ That points out the two‐phonon Raman relaxation pathway to be the mechanism limiting the stability of the magnetic moments of the studied SMMs on surfaces, and it explains the enhanced SMM behavior of the molecules adsorbed on an oxide film.

The hybridization between molecular and substrate orbitals, which is expected to be slightly stronger on the Ag surface than on MgO, might play a role in the sense of a stronger coupling of the molecules to the substrate phonons. Furthermore, it could lead to a modification of the ligand electronic structure, which could cause a departure from the ideal *D*_4d_ symmetry, thus promoting QTM. However, as stated before, the shrinking of the magnetic hysteresis on TbPc_2_/Ag compared to MgO cannot be reproduced by a strong increase of the QTM rate. In view of this observation and taking into account the shielded nature of the 4f shell, which is well protected from hybridization with the substrate, it appears that the effect of molecule‐substrate hybridization is limited to the first aspect of stronger coupling to the substrate phonons.

## 4 Conclusion

In summary the relevant magnetization relaxation mechanisms of LnPc_2_ SMMs on an oxide and on a metallic surface were determined to unravel the role of the oxide film in enhancing the SMM's magnetic stability. The study reveals that the MgO‐induced deceleration of the SMMs' magnetization dynamics are due to the suppression of the two‐phonon Raman relaxation pathway. The observation of the enhanced magnetic stability for two representatives of the LnPc_2_ family suggests a general stabilizing effect also for other SMMs. This work highlights the influence of spin‐phonon coupling on the magnetic stability of surface adsorbed SMMs. It suggests that the use of weakly adsorbing substrates with low phonon density of states is beneficial for the construction of molecule‐based spintronic devices and for a further increase of the blocking temperature of surface adsorbed SMMs.

## 5 Experimental Section

*Sample Preparation*: The Ag(100) single crystal was cleaned prior to MgO deposition by repeated cycles of Ar^+^ ion sputtering and annealing at 740 K. The MgO film was deposited by thermal sublimation of Mg (*T*_Mg_ ≈ 680 K) in an O_2_ partial pressure of 10^−6^ mbar onto the Ag(100) crystal held at a temperature of 633 K. The DyPc_2_ molecules were sublimed from a Knudsen cell at a temperature of 658 K onto the substrate held at room temperature. The sublimation rate of the DyPc_2_ molecules was determined using a quartz microbalance. The MgO film thickness was characterized by XAS at the Mg K edge. The preparation of the TbPc_2_/MgO/Ag(100) and TbPc_2_/Ag(100) systems is described in ref. 30.

*X‐Ray Absorption Measurements*: The XAS measurements were performed in total electron yield (TEY) mode at the X‐Treme beam line at the Swiss Light Source, Paul Scherrer Institute, Switzerland.^59^ The XAS signal is defined as the sum of the two corresponding polarized X‐ray spectra, i.e., XAS = (μ^+^ + *μ^−^*) whereas XLD and XMCD are the differences, i.e., XLD = (μ_v_ – μ_h_) and XMCD = (μ^+^ − *μ^−^*). The in situ sample preparation was performed at the X‐Treme beamline's preparation environment under ultra‐high vacuum conditions (*p*_0_ ≈ 10^−10^ mbar). The X‐ray beam was impingent at normal (θ = 0°) or grazing (θ = 60°) incidence with respect to the sample surface, while the magnetic field was always collinear with the beam propagation direction. The X‐ray spot size at the sample position was 1.2 × 0.3 mm^2^ and the flux was kept low (ϕ = 0.05 ph nm^−2^ s^−1^) to avoid beam damage and X‐ray induced demagnetization effects. No spectral changes over time were observed indicating the absence of beam damage. The temperature at the sample surface was 2.5 K ± 0.5 K. Slightly different temperatures within this error may arise from, e.g., small differences in mounting of the Ag single crystals on the sample plates and in the resulting differences in thermal coupling. The X‐ray spectra were normalized by the pre‐edge value. The XLD and XMCD spectra were normalized to the sum of the main Dy M_5_ peak (≈ 1290.05 eV) amplitudes of the two polarizations. A linear background was subtracted.

*Magnetization Dynamics Calculations and Fits*: Equation (1) was solved numerically using the following form with discrete time steps Δ*M*  = −Γ(*H*(*t*),*T*)[*M*(*H*(*t*)) − *M*_eq_ (*H*(*t*),*T*)]Δ*t* The time evolution of the magnetization after a single time step is thus given by *M* (*t* + Δ*t*) = *M*(*t*) + Δ*M*. A time step of Δ*t* = 0.1 ms was used. In this way the whole *M*(*H*) can be obtained starting with a given initial condition *M*(*t* = 0). The equilibrium magnetization *M*_eq_(*H,T*) was calculated using a spin‐Hamiltonian approach (see the corresponding paragraph in the Experimental Section) taking into account the Stevens parameters reported in the literature for the anionic LnPc_2_.^44^ The experimental protocol was reproduced in the simulations, with the initial condition of *H*(*t* = 0) = 6.8 T and *M*(*t* = 0) = *M*_eq_(*H* = 6.8 T). The experimental field sweep rate of d*H*/d*t* = 2 T min^−1^ was taken into account. Also, a smoothing of the simulated curves by adjacent averaging across a width of δ = 0.1 T was applied in analogy to the treatment of the experimental data. An interruption time of the field sweep of 20 s was taken into account at 0 T reflecting the time required for the magnet power supply to change the magnetic field polarity.

*Density‐Functional Calculations*: The relaxed YPc_2_ molecular structure on the MgO/Ag(100) and Ag(100) substrates was calculated via density functional theory within the Kohn–Sham formalism^60^ and with use of QuickStep module^61^ in the CP2K code (rB86‐vdW‐DF2 approximation^62^). More details are given in the Supporting Information.

*Spin‐Hamiltonian Calculations*: The equilibrium magnetization *M*(*H*) shown in Figure 5 was calculated using a spin‐Hamiltonian model implemented in a MATLAB code operating on the lowest manifolds *J* = 15/2 for Dy^3+^ and *J* = 6 for Tb^3+^. The ligand field was taken into account using Stevens operators, with the Stevens parameters obtained from ref. 44 after conversion by α, β, and γ factors.^48^ The Hamiltonian was solved by full diagonalization and magnetization was calculated considering thermodynamical population of the energy levels.

## Conflict of Interest

The authors declare no conflict of interest.

## Supporting information

Supplementary
