# Supplementary material for: Understanding the Superior Stability of Single‐Molecule Magnets on an Oxide Film
Source: Adv Sci (Weinh). 2019 Sep 30;6(22):1901736. doi: 10.1002/advs.201901736 (PMC6864999; doi:10.1002/advs.201901736)
Supplement: Supplementary file 1 — Supplementary [file ADVS-6-1901736-s001.pdf]

Copyright WILEY-VCH Verlag GmbH & Co. KGaA, 69469 Weinheim, Germany, 2019.

Supporting Information

for *Adv. Sci.,* DOI: 10.1002/advs.201901736

Understanding the Superior Stability of Single-Molecule Magnets on an Oxide Film

Michał Studniarek, Christian Wäckerlin, Aparajita Singha, Romana Baltic, Katharina Diller, Fabio Donati, Stefano Rusponi, Harald Brune, Yanhua Lan, Svetlana Klyatskaya, Mario Ruben,* Ari Paavo Seitsonen, and Jan Dreiser**

Copyright WILEY-VCH Verlag GmbH & Co. KGaA, 69469 Weinheim, Germany, 2019.

Supporting Information

Understanding the Superior Stability of Single-Molecule Magnets on an Oxide Film

Michał Studniarek, Christian Wäckerlin, Aparajita Singha, Romana Baltic, Katharina Diller, Fabio Donati, Stefano Rusponi, Harald Brune, Yanhua Lan, Svetlana Klyatskaya, Mario Ruben*, Ari P. Seitsonen, Jan Dreiser**

Dr. M. Studniarek, Dr. J. Dreiser
Swiss Light Source, Paul Scherrer Institut (PSI), CH-5232 Villigen PSI, Switzerland. E-mail: jan.dreiser@psi.ch

Dr. C. Wäckerlin, Dr. A. Singha, Dr. R. Baltic, Dr. K. Diller, Prof. F. Donati, Dr. S. Rusponi, Prof. H. Brune, Dr. J. Dreiser
Institute of Physics (IPHYS), École Polytechnique Fédérale de Lausanne (EPFL), Station 3, CH-1015 Lausanne, Switzerland.

Dr. C. Wäckerlin
Institute of Physics, The Czech Academy of Sciences, Cukrovarnická 10, 162 00 Prague 6, Czech Republic.

Dr. A. Singha, Prof. F. Donati
Center for Quantum Nanoscience, Institute for Basic Science (IBS), 03760 Seoul, Republic of Korea and Department of Physics, Ewha Womans University, Seoul 03760, Republic of Korea.

Dr. Y. Lan, Dr. S. Klyatskaya, Prof. M. Ruben
Institute of Nanotechnology (INT), Karlsruhe Institute of Technology (KIT), Hermann-von-Helmholtz-Platz 1, D-76344, Eggenstein-Leopoldshafen, Germany.

Prof. M. Ruben
Institut de Physique et Chimie des Matériaux de Strasbourg (IPCMS), CNRS, Université de Strasbourg, 23 rue du Loess, BP 43, F-67034 Strasbourg Cedex 2, France.

Prof. A. Seitsonen
Département de Chimie, École Normale Supérieure, F-75005 Paris, France, and CNRS, Paris Sciences et Lettres, Sorbonne Université, F-75005 Paris, France.

Contents

1. Details of DFT calculations 2
2. X-ray linear dichroism (full energy range of spectra in Figure 2). 4
3. X-ray linear dichroism of the sample used to acquire magnetic hysteresis loop presented in Figure 4a 5
4. X-ray magnetic circular dichroism of $DyPc_2$/Ag(100) 5
5. Relaxation rate of the Orbach process for $DyPc_2$ and $TbPc_2$ on MgO/Ag and Ag surfaces 6
6. Simulation of magnetization dynamics in the $TbPc_2$/MgO system including field dependence of Raman relaxation rate $\sim C|H|^l$ with l = 1, 2, 3, 4 7
7. Changes of $TbPc_2$ magnetic hysteresis when switching from MgO to Ag surface upon varying QTM and direct spin-phonon (S-P) relaxation rates 8

1. Details of DFT calculations

We performed total energy calculations using density functional theory (DFT)[1] within the Kohn-Sham formalism[2] using the QuickStep module[3] in the CP2K code (http://www.CP2K.org/). The rB86-vdW-DF2 approximation[4] to the exchange-correlation functional was applied. We used DZVP-MOLOPT-SR-GTH basis sets and a cut-off energy of 1300 Ry and the relative cut-off energy 70 Ry to expand the Kohn-Sham orbitals and the augmented electron density, respectively. The pseudo potentials were of the type Goedecker-Teter-Hutter.[5] 2 × 2 points were used in the calculations due to the relatively large dimensions, together with the Fermi-Dirac broadening of the occupation numbers with a width of 25.8 meV. The molecule was inserted in the experimental super-cell of MgO/Ag(100), which contains 25 atoms per layer. Five layers of MgO were employed. The experimental lattice constant of Ag of 4.09 Å was used in the calculations.[6] Six layers of Ag atoms in the Ag(100) support were included, and three top layers of the metal were relaxed both in the calculations of YPc_2/MgO/Ag(100) and YPc_2/Ag(100), with all the atoms relaxed in the MgO (when present) and the adsorbate.

Table S1. Adsorption energies of YPc_2 on the Ag(100) hollow site and on the MgO(5 ML)/Ag(100) oxygen-on-top and magnesium-on-top sites.

	E_b [eV]	
Adsorption site	MgO(5ML)	Ag(001)
O_{ot}-R1	4.786	
Mg_{ot}-R1	4.228	
hollow		4.917

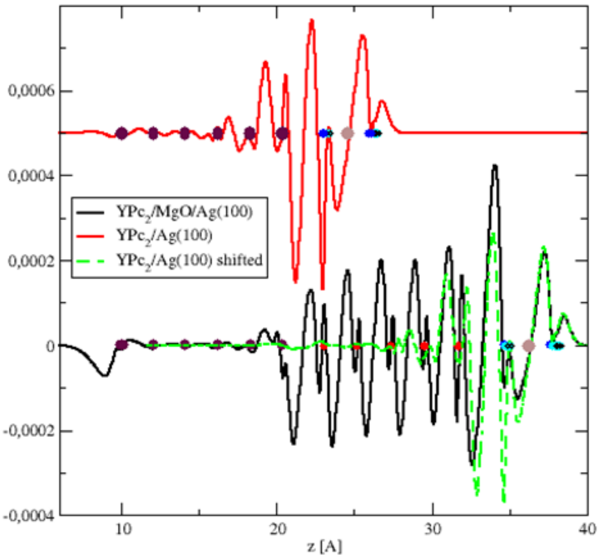

Figure S1. Charge transfer between YPc_2 and surfaces. Differences of electronic density $\Delta n(z) = n_{YPc2/Ag} - n_{YPc2} - n_{Ag}$ (red curve) and $\Delta n(z) = n_{YPc2/MgO/Ag} - n_{YPc2} - n_{MgO/Ag}$ (black curve) as explained in the text of the SI. The *z* direction denotes the surface normal, and the plotted densities were averaged in the surface (*x,y)* plane. The green dashed curve has been offset horizontally to match the position of the Y(III) ions. The red curve has been offset vertically for clarity.

In order to evaluate the transfer of charge between $LnPc_2$ molecules and the substrate we have calculated the differences of electronic density $\Delta n(\mathbf{r}) = n_{YPc2/MgO/Ag} - n_{YPc2} - n_{MgO/Ag}$ and $\Delta n(\mathbf{r}) = n_{YPc2/Ag} - n_{YPc2} - n_{Ag}$. Here, $n_{YPc2/MgO/Ag}$ is the electron density of the full system, *i.e.*, YPc_2 adsorbed on 5 MLs of MgO/Ag(100), and n_{YPc2} and $n_{MgO/Ag}$ are the densities of the molecule and the substrate, calculated separately with the coordinates of the full system.

In Figure S1 one-dimensional plots of $\Delta n(z)$ along the surface normal direction z averaged in the (x,y) plane are shown. The green, shifted, curved is aligned so that the yttrium(III) ion is at the same value of z, to clarify the differences between the two curves around the molecule at the right of the plot. The brown circle is the yttrium(III) ion, the cyan and the blue circles are the two phthalocyanine molecules. The red curve has been offset vertically for clarity.

The results plotted in Figure S1 indicate that there are significant movements of charges toward the YPc_2 molecules in both cases of Ag(100) and MgO/Ag(100). Interestingly, in both cases there is a depletion, *i.e.*, a dip, at the Ag(100) surface and there are peaks just below both phthalocyanine ligands. The pattern looks similar for both substrates in the vicinity of the molecules. Since in the case of $TbPc_2$/Ag(111) a charge transfer between molecules and surface was found leading to the absence of the ligand hole (radical spin),[7,8] we interpret these results in that the ligand hole is absent in YPc_2/MgO(5 ML)/Ag(100), too.

2. X-ray linear dichroism (full energy range of spectra in Figure 2).

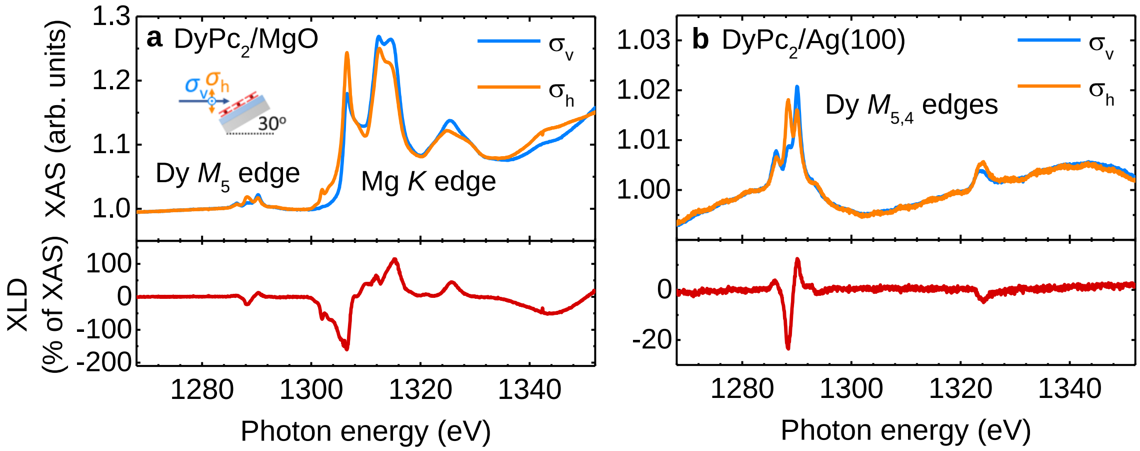

Figure S2. Full scale X-ray linear dichroism spectra of $DyPc_2$. X-ray absorption spectra recorded with linear vertical (σ_v) and horizontal (σ_h) X-ray polarization (top panels) and corresponding XLD (bottom panels) at the Dy $M_{4,5}$ edges of $DyPc_2$ (sub-ML) on (a) MgO(4 ML)/Ag(100) and on (b) Ag(100). The spectra were recorded at grazing X-rays incidence 60° to sample normal, at $T = 2.5 \pm 0.5$ K and at 50 mT of applied external magnetic field.

3. X-ray linear dichroism of the sample used to acquire magnetic hysteresis loop presented in Figure 4a

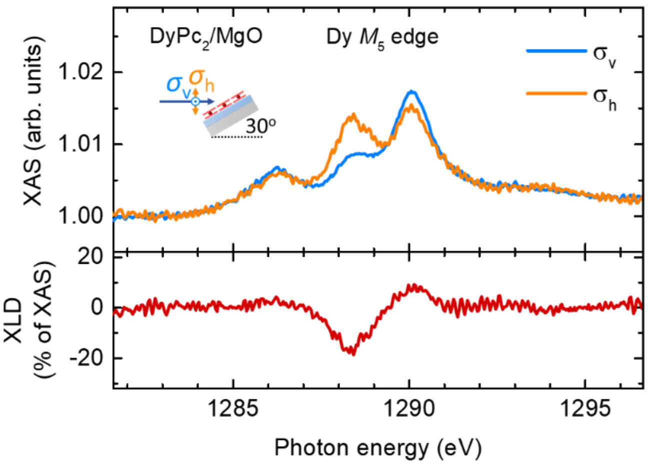

Figure S3. X-ray linear dichroism of the sample from Figure 4. X-ray absorption spectra recorded with linear vertical (σ_v) and horizontal (σ_h) X-ray polarization (top panel) and corresponding XLD (bottom panel) at the Dy M_5 edge of $DyPc_2$ (sub-ML) on MgO(4 ML)/Ag(100). The spectra were recorded at $T = 2.5 \pm 0.5$ K, at grazing X-ray incidence of 60° to the sample normal, and at 50 mT of applied external magnetic field.

4. X-ray magnetic circular dichroism of $DyPc_2$/Ag(100)

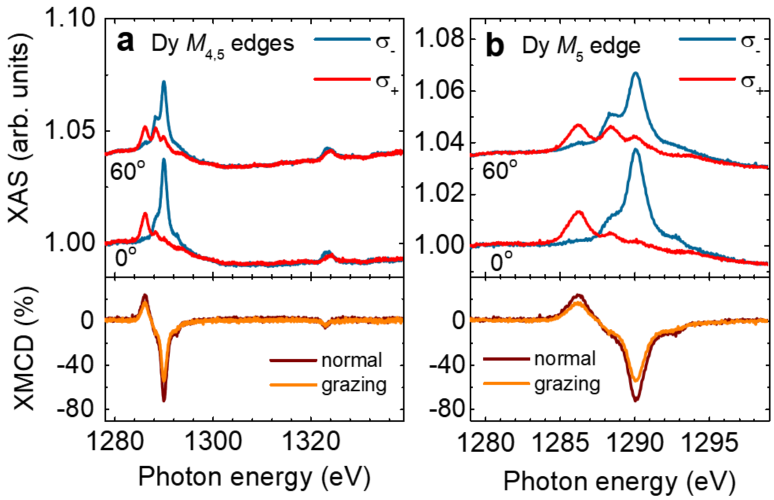

Figure S4. X-ray linear dichroism of $DyPc_2$/Ag(100). XAS (top panels) and corresponding XMCD (bottom panels) acquired on $DyPc_2$(sub-ML)/Ag(100) at (a) Dy $M_{4,5}$ edges of $DyPc_2$ with (b) zoom on Dy M_5 edge only. The spectra were recorded at $T = 2.5 \pm 0.5$ K at normal (0°) and grazing (60°) X-ray incidence and at 6.8 T of applied magnetic field.

5. Relaxation rate of the Orbach process for $DyPc_2$ and $TbPc_2$ on MgO/Ag and Ag surfaces

In the Orbach process one phonon is absorbed promoting the Ln(III) ion to an excited state, and subsequently another phonon is emitted, with the energy difference of the phonons equal to the Zeeman energy. The rate is given by the Arrhenius-type law:[9]

$$\Gamma_{\mathrm{Orb}} = \tau_{\mathrm{o}}^{-1}\exp(-\Delta/k_{\mathrm{B}}T)$$

where τ_{o}^{-1} is the attempt frequency and Δ denotes the effective energy barrier for magnetization reversal. To establish the relaxation rate of the Orbach process we used values from Reference [9], i.e., $\Delta = 3.84$ meV, $\tau_{\mathrm{o}} = 3.3\cdot10^{-6}$ s for $DyPc_2$ and $\Delta = 0.032$ eV, $\tau_{\mathrm{o}} = 2\cdot10^{-8}$ s for $TbPc_2$. This leads to the Orbach process relaxation rates of $1.6\cdot10^{-57}$ s^{-1} for $TbPc_2$ at 2.5 K, $5.5\cdot10^{-3}$ s^{-1} for $DyPc_2$ at 2.5 K. These relaxation rates insignificantly contribute to the magnetization dynamics of studied molecules, nevertheless, they are taken into account in the simulation. The Orbach process rates are not plotted in Figure 5 in the main text for clarity.

6. Simulation of magnetization dynamics in the $TbPc_2$/MgO system including field dependence of Raman relaxation rate ~$C|H|^l$ with l = 1, 2, 3, 4

In the main text we explain that the theoretical value of the l exponent in the magnetic field dependence of Raman relaxation rate ~$|H|^{l,}$ remains unknown and differs between various literature reports. We have tested how well the simulation matches the experimental data for integer values of l , $0 \leq l \leq 4$. The case of $l = 0$, *i.e.*, field-independent Raman relaxation yields the best fit and is presented in the main text. We find that, in addition, when including 20% of fast relaxing molecules, as described in the main text, only $l = 1$ and 2 provide reasonable fits to the experimental data (Figure S5). Further increasing the l parameter yields a strong deviation of the fit in the low-field range. Therefore, our model implies that the relaxation rate ascribed to the Raman process in the studied systems is still consistent with a field dependence ~H^l with $0 \leq l \leq 2$.

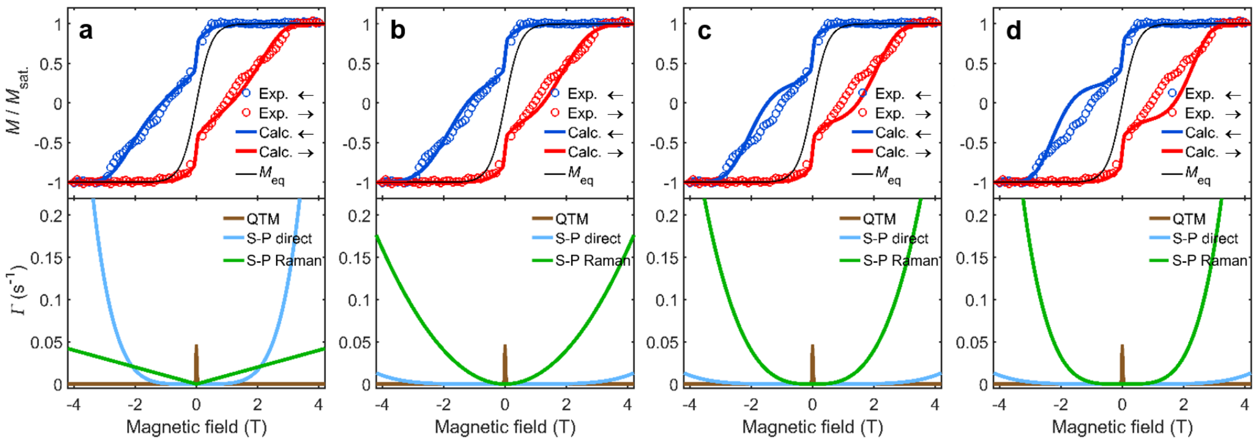

**Best fit parameters:**	**Best fit parameters:**	**Best fit parameters:**	**Best fit parameters:**
$C_{l=1}$ = 0.01·$|H|$ s^{-1}	$C_{l=2}$ = 0.01·H^2 s^{-1}	$C_{l=3}$ = 0.005·H^2 s^{-1}	$C_{l=4}$ = 0.002·H^4 s^{-1}
A = 5·10^4 T^{-5} s^{-1}	A = 1·10^5 T^{-5} s^{-1}	A = 1·10^5 T^{-5} s^{-1}	A = 1·10^5 T^{-5} s^{-1}
B_1 = 0.045 s^{-1}	B_1 = 0.045 s^{-1}	B_1 = 0.045 s^{-1}	B_1 = 0.045 s^{-1}
B_2 = 1.5·10^4 T^{-2}	B_2 = 1.5·10^4 T^{-2}	B_2 = 1.5·10^4 T^{-2}	B_2 = 1.5·10^4 T^{-2}

Figure S5. Magnetization dynamics modeling using field-dependent Raman relaxation. (Top panels) Experimental and best-fit calculated magnetic hysteresis loops for $TbPc_2$(sub-ML)/MgO(5 ML)/Ag(100). The experimental data is taken from Reference [6]. (Bottom panels) The contributions to the total relaxation rate of the relevant relaxation processes used in the best-fit calculation including field-dependent Raman rate as (a) ~$|H|$, (b) ~H^2, (c) ~$|H|^3$and (d) ~H^4. The field sweep rate was 2 T/min in both experiment and calculation.

7. Changes of $TbPc_2$ magnetic hysteresis when switching from MgO to Ag surface upon varying QTM and direct spin-phonon (S-P) relaxation rates.

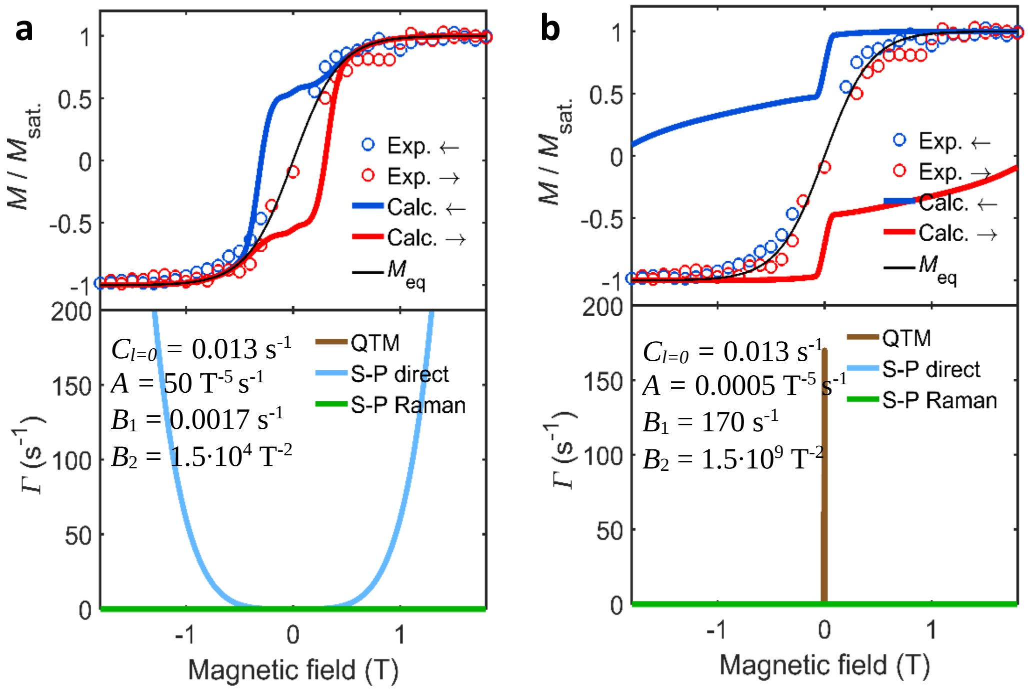

Figure S6. Magnetization dynamics modeling of $TbPc_2$ on Ag(100). Changes to the magnetic hysteresis of $TbPc_2$/Ag as induced by increasing (a) 10^5 times the QTM rate or (b) 10^5 times the spin-phonon direct relaxation rate while keeping the other relaxation rates equal to the ones found for $TbPc_2$/MgO/Ag(100) as listed in Table 1 of the main text.

REFERENCES

[1] P. Hohenberg, W. Kohn, *Phys. Rev.* **1964**, *136*, B864.

[2] W. Kohn, L. J. Sham, *Phys. Rev.* **1965**, *140*, A1133.

[3] J. VandeVondele, M. Krack, F. Mohamed, M. Parrinello, T. Chassaing, J. Hutter, *Comput. Phys. Commun.* **2005**, *167*, 103.

[4] I. Hamada, *Phys Rev B* **2014**, *89*, 121103.

[5] S. Goedecker, M. Teter, J. Hutter, *Phys Rev B* **1996**, *54*, 1703.

[6] C. Wäckerlin, F. Donati, A. Singha, R. Baltic, S. Rusponi, K. Diller, F. Patthey, M. Pivetta, Y. Lan, S. Klyatskaya, M. Ruben, H. Brune, J. Dreiser, *Adv. Mater.* **2016**, *28*, 5195.

[7] F. Ara, Z. K. Qi, J. Hou, T. Komeda, K. Katoh, M. Yamashita, *Dalton Trans.* **2016**, *45*, 16644.

[8] J. Hellerstedt, A. Cahlík, M. Švec, B. de la Torre, M. Moro-Lagares, T. Chutora, B. Papoušková, G. Zoppellaro, P. Mutombo, M. Ruben, R. Zbořil, P. Jelinek, *Nanoscale* **2018**, *10*, 15553.

[9] N. Ishikawa, M. Sugita, T. Ishikawa, S. Y. Koshihara, Y. Kaizu, *J. Phys. Chem. B* **2004**, *108*, 11265.
